# Health insurance and the financial implications of sickle cell disease among parents of affected children attending a tertiary facility in Lagos, south-west Nigeria

**DOI:** 10.11604/pamj.2020.36.227.24636

**Published:** 2020-07-29

**Authors:** Chibuzor Franklin Ogamba, Adeseye Michael Akinsete, Henry Somtochukwu Mbaso, Oluwagbemiga Ayomiposi Adesina

**Affiliations:** 1 Department of Pediatrics, Lagos University Teaching Hospital, Lagos, Nigeria,; 2 Department of Pediatrics, Faculty of Clinical Sciences, College of Medicine, University of Lagos/Lagos University Teaching Hospital, Lagos, Nigeria,; 3 Faculty of Clinical Sciences, College of Medicine University of Lagos, Nigeria

**Keywords:** Children, cost, health insurance, sickle cell disease, Nigeria

## Abstract

**Introduction:**

there is a paucity of data on the financial implications of sickle cell disease on households of affected children and their use of health insurance in Nigeria. This study assessed the awareness of health insurance, patterns of health service utilization and financial implications of sickle cell disease among children seeking care at a tertiary facility in Nigeria.

**Methods:**

a structured questionnaire was administered to parents of 314 children with sickle cell disease attending the pediatric hematology unit of the Lagos University Teaching Hospital between May and December 2019.

**Results:**

mean age of the children was 91.5 ± 43.1 months. M: F was 1.17: 1. 45.5% of households earned above NGN 150,000 (USD 417) monthly. 71.3% of the parents had heard of health insurance but only 20.7% were enrolled in a health insurance scheme. Awareness of health insurance was significantly associated with social class (p=0.000) and monthly household income (p=0.000). 60.8% of the parents preferred pre-facility treatment. Social class (p=0.01) and monthly household income (p=0.001) were significantly associated with home treatment. Time on admission ranged from 2-18 days with an average of 4.31 days. Average cost of hospitalization was USD 148 ± USD 14.2 and total cost of care incurred was USD 20,787. Neither age of child (p=0.857), estimated household income (p=0.863) nor social class (p=0.397) was associated with cost of care.

**Conclusion:**

a high cost of care was observed in our study population underscoring the need for increased awareness and access to health insurance for households of children with sickle cell disease.

## Introduction

Sickle cell disease is the commonest inherited genetic disorder of haemoglobin in Africa with about 180,000 new births annually of the global 330,000 [1]. Globally, Nigeria has the highest burden of sickle cell disease occurring in about 2%-3% of new-borns [2]. It is a chronic disorder characterized by recurrent vaso-occlusive crises requiring frequent hospitalizations. Thus, it constitutes a financial stress because of repeated hospital admissions [3]. Following the world health assembly resolution in 2005 which urged member states to transition to universal health coverage [4], Nigeria implemented the National Health Insurance Scheme (NHIS), a social health insurance policy. Nevertheless, as at 2017, domestic private healthcare expenditure and out of pocket personal expenditure were still 77.2% each of current health expenditures [5]. These forms of healthcare financing have been shown to create health disparities between the rich and the poor and are associated with catastrophic health expenditures, a situation in which households and individuals compromise on other basic needs in order to maintain healthcare costs [6,7]. Studies have reported gaps in knowledge and utilization of health insurance even among professionals [8].

Though some studies have been done on household health expenditures in Nigeria [3,9-11], to the best of our knowledge, only a few focused on the cost implications of sickle cell disease in Nigeria [3,7,12] and examined the perception towards health insurance among these households. In mitigating the barriers out-of-pocket expenditure poses in the prompt and efficient management of patients with sickle cell disease, health systems need to constantly evaluate the financial implications of sickle cell disease management on affected households in order to efficiently implement cost effective measures geared towards improving access to care and reduction of mortality [12]. Bearing in mind that knowledge and favourable dispositions towards health insurance may affect utilization, households affected need to know about health insurance, understand its benefits and be able to access health insurance packages for their children. Therefore, we assessed the perception and use of health insurance, pattern of health service utilisation and expenditures on healthcare services among parents of children with sickle cell disease receiving care at the Lagos University Teaching Hospital and made recommendations concerning financing of their healthcare.

## Methods

**Study design and setting:** this was a hospital-based cross-sectional study of parents of children with SCD receiving care at the Lagos University Teaching Hospital (LUTH) in Lagos, Nigeria, which assessed their awareness and use of health insurance, pattern of health service utilisation and their expenditures on healthcare from May 2019 to December 2019.

**Study participants:** parents who gave consent were consecutively enrolled in the study.

**Data collection:** a standardized questionnaire adapted from a review of similar studies [6,7,12] was completed for each participant. The standardized questionnaire consisted of the following sections: section A assessed the socio-demographic details of the child, parents´ occupation and educational attainments, family income, source of health financing and number of other siblings in the household. The household of each child was then assigned into a social class with a classification proposed by Oyedeji [13] using the parents´ occupation and educational levels grouped as 1-5. For each parent´s educational level, class 1 was assigned to parents with a University degree or equivalent, class 2 to those with school certificate in addition to teaching or other professional training, class 3 to those with school certificates only, grade II teacher´s certificate or equivalent, class 4 to those with first school leaving certificates and class 5 to those who did not have any of the above. For each parent´s occupation, class 1 was assigned to parents who were senior public servants, professionals (e.g. doctors, lawyers etc.), managers, large scale traders, business men and contractors, class 2 to those who were intermediate grade public servants or professionals, class 3 to those who were junior school teachers and professionals and artisans, class 4 to those who were semi-skilled (e.g. ward maids, petty traders etc.) and class 5 to those who were either unemployed or unskilled (e.g. cleaners, house helps etc.). Scores for both parents were then added up and divided by 4 and approximated to the nearest whole.

The five classes were subsequently sub-classified into upper class (I and II), middle class (III) and lower class (IV and V). Also, the estimated monthly household income as well as respondents´ source of funds to make payments at the health facility was assessed. The estimated monthly household income was assessed by asking parents to estimate total of salaries and all other traceable sources of income. Section B assessed parents´ perception and use of health insurance, pattern of health service utilisation prior to presentation and method of payment for services. Section C assessed illnesses and treatments given to each child with SCD during the event as well as amounts spent. These amounts included the hospital consultation fees, admission fees, cost of investigations, cost of drugs prescribed, cost of procedures performed and other user charges. Direct questioning and inspection of receipts were used to derive cost estimates filled into the standardized questionnaire. The diagnoses of illnesses were made based on clinical information and laboratory tests where necessary and the duration of hospital admission was calculated from admission to discharge for those on in-hospital admissions. Also, the costs of transportation, foods and other expenditures that are often not receipted were excluded.

**Determination of sample size:** a total of 314 respondents were recruited for the study. This sample size was calculated using the modified Cochran formula for sample size calculation in smaller populations at a significance level of 95%, with p=0.5 for maximum variability and an alpha level of 0.05. A total of 1,710 was used as the population size and represents the total number of sickle cell disease patients managed by the unit in the previous year; 1,360 out-patients and 350 in-patients.

**Data analysis:** data was entered and coded using Microsoft Excel 2007 and subsequently analysed electronically using SPSS statistical software version 20. Data was presented as means, frequencies and percentages. Chi-square tests were used to determine socio-economic correlates of awareness of health insurance and preference for home treatment. One-way ANOVA and independent t-tests were used to describe the effects of socio-economic variables and duration of hospitalization on cost of care. P value was set at less than 0.05 at 95% confidence interval.

**Ethics:** approval for this study was obtained from the health research and ethics committee of the Lagos University Teaching Hospital. The participants were informed about the significance of the study and how honest and fair answers were important when answering the standardized questionnaire. Consent was obtained before completion of the standardized questionnaire.

## Results

**Socio-demographic characteristics of the respondents:** three hundred and fourteen households were recruited into the study, 222 children were recruited from routine clinic follow-up visits, while 92 children were recruited from in-hospital admissions. The study participants were aged between 5-193 months with a mean age of 91.5 ± 43.1 months. The number of other siblings in the household ranged from 0-5 with a median number of 2. Majority (53.8%, n=169) of the children were males and had parents who were christian (69.7%, n=219) and were married (92.0%, n=289). Majority of the households were in the upper social class (67.5%, n=212) and had a monthly income greater than USD 417/NGN 150,000 (45.5%, n=143). Nine households earned at or below the national minimum wage (USD 50/NGN 18,000) during the study period (Table 1). To make payments at the health facility, 300 (95.5%) households used personal savings, 55 (17.5%) households used contributions from friends and family members, 37 (11.8%) households used health insurance and 30 (9.6%) households used monies borrowed from friends and family members.

**Table 1:** study sample characteristics

Characteristic	Frequency (%) or Mean ± SD^c^ [Range]
**Age of child (months)**	**91.5 ± 43.1 [5 - 193]**
**Sex**	
Male	169 (53.8)
Female	145 (46.2)
**Social Class**	
Upper (I and II)	212 (67.5)
Middle (III)	65 (20.7)
Lower (IV)	37 (11.8)
**Number of other children in household**	
0	21 (6.7)
1-2	211 (67.2)
3-5	82 (26.1)
**Religion of parents**	
Christianity	219 (69.7)
Islam	95 (30.3)
**Marital Status^a^**	
Single	13(4.1)
Married	289(92.0)
Separated/Widowed	11 (3.5)
Estimated household monthly income*^a^	
≤USD139	61 (19.4)
USD139-USD250	44 (14.0)
USD250-USD417	61 (19.4)
>USD417	143 (45.5)
Households earning less than/equal to National minimum wage^b^	9 (2.9)
**Enrolled in Health Insurance**	
Yes	65 (20.7)
No	249 (79.3)
^*^1 US Dollar = N360 during study period ^a^Missing data	^b^Minimum wage = N18,000 (USD50) ^c^SD = Standard Deviation

**Health insurance and patterns of health service utilisation:** two hundred and twenty-four (71.3%) parents had heard of health insurance but only 65 (20.7%) were enrolled in a health insurance scheme. Among enrollees, type of insurance scheme payment was either by themselves (2.9%, n=9), by government e.g. National Health Insurance Scheme (7.0%, n=22) or by their employers (10.5%, n=33). Majority of the parents (68.5%, n=215) believed health insurance was valuable, however, 140 (44.6%) households were willing to enrol in a health insurance scheme.

**Socio-economic correlates of awareness of health insurance:** awareness of health insurance was significantly associated with social class (p=0.000) and monthly household income (p=0.000). There was more awareness of health insurance among parents in higher social classes and awareness decreased with decreasing social class. There was also more awareness of health insurance among families who earned above USD 417/NGN 150,000 than among those who earned ≤USD 417/NGN 150,000 (Table 2).

**Table 2:** socio-economic correlates of awareness of health insurance and home treatment

	Awareness of health insurance			Home treatment		
Characteristic	Yes	No	P value	Yes	No	P value
**Age of child (months)**						
<60	48	22		38	32	
60-120	131	44	0.36	111	63	0.64
>120	46	23		43	26	
**Sex**						
Male	125	44		105	63	
Female	100	45	0.19	87	58	0.85
**Social Class**						
Upper (I and II)	180	32		116	95	
Middle (III)	33	32	0.00	47	18	0.01
Lower (IV)	12	25		29	8	
**Number of other children in household**						
0	17	4		10	11	
1-2	157	54	0.07	126	84	0.31
3-5	51	31		56	26	
**Religion of parents**						
Christianity	163	56		132	87	
Islam	62	33	0.07	60	34	0.30
**Marital Status**						
Single	8	5		10	3	
Married	210	79	0.63	175	113	0.47
Separated/Widowed	7	4		6	5	
Estimated household monthly income*						
≤USD139	32	29		39	22	
USD139-USD250	23	21		32	12	
USD250-USD417	46	15	0.00	47	14	0.001
>USD417	122	21		70	72	

* 1 US Dollar = N360 during study period

**Socio-economic correlates of home treatment:** most parents (60.8%, n=191) indicated preference to treat their child at home before seeking care at the health facility. When asked for possible reasons, responses included that the illness is not serious (47.1%, n=148), that they knew what medicines to give their children (41.4%, n=130), that it was cheap to do so (13.1%, n=41), long distance to the healthcare facility (3.2%, n=10), that chemists were nearer (1.9%, n=6), that they make use of native medications e.g. “Agbo” (1.0%, n=3), that health workers are unfriendly (1.0%, n=3), that health workers are not readily available at the health facility (0.6%, n=2) and religious/traditional beliefs (0.6%, n=2). Social class and monthly household income were significantly associated with decision to treat child at home (p=0.01 and 0.001 respectively) (Table 2).

**Patterns of clinical presentation and associated comorbidities:** of the in-hospital admissions, 49 (51.0%) participants had exchange blood transfusions and 35 (36.5%) of them were part of the chronic transfusion program for the prevention of stroke. The most frequently occurring comorbidities were vaso-occlusive crisis (26.0%, n=25), cerebrovascular accident (9.4%, n=9) and acute chest syndrome (7.3%, n=7). Other comorbidities are listed and participants also had more than one comorbid condition (Table 3). Time on admission ranged from 2-18 days with an average of 4.31 days.

**Table 3:** associated comorbidities among hospitalized patients

Comordity	Frequency (%)
Vaso-occlusive crises	25 (26.0)
Cerebrovascular accident	9 (9.8)
Acute Chest Syndrome	7 (7.6)
Priapism	4 (4.3)
Malaria	4 (4.3)
Bronchopneumonia	3 (3.3)
Severe Anemia	2 (2.2)
Anemic heart failure	1 (1.1)
Osteomyelitis	2 (2.2)
Leg Ulcer	2 (2.2)
Peri-orbital cellulitis	1 (1.1)
Lobar Pneumonia	1 (1.1)
Sequestration crisis	1 (1.1)
Asthma	1 (1.1)
Gastroenteritis	1 (1.1)
Umbilical Hernia	1 (1.1)
Sepsis	1 (1.1)
Chronic transfusion therapy for Stroke prevention	35 (38.0)

* Some children had more than one comorbidity, N=92

**Pattern of health expenditures and cost of hospitalization:** total amount spent by parents on outpatient visits ranged from NGN530 to NGN31, 530 (USD1.47 - USD87.58) with an average cost of NGN9,426.76 ± NGN481.06 (USD26.19 ± USD1.34) and expenditures covered registration/consultation fees, cost of drugs and investigations carried out. Total amount spent by parents on in-hospital admissions ranged from NGN15,530 to NGN276, 530 (USD87.58 to USD768.14) with an average total cost of NGN59,237.47 ± NGN5,094.96 (USD147.88 ± USD14.15) and expenditures covered registration fees, admission fees, cost of drugs and investigations, cost of procedures performed and miscellaneous in-hospital expenditures. The overall health expenditure ranged from NGN530 to NGN276, 530 (USD1.47 to USD768.14) with a mean of NGN23, 908.47 ± NGN35,086.57 (USD66.41 ± USD97.46) per household. Majority of the expenditures were made on hospital utilities which include consultation/registration fees, admission fees, cost of procedures and hospital miscellaneous expenses including blood transfusion services, nursing services and other service charges.

Total expenditure made by the respondents was NGN7, 483, 350 (USD20,787) (Table 4). Average cost of hospitalization for patients with vaso-occlusive crisis was NGN40,875.42 ± NGN20,380.98 (USD113.54 ± USD56.61) and expenditures ranged from NGN15,530 to NGN84,530 (USD87.58 to USD234.81). Average cost of hospitalization for patients who were on the chronic transfusion program due to high risk of stroke was NGN44,864.29 ± NGN8,874.19 (USD124.62 ± USD24.65) and expenditures ranged from NGN9,750 to NGN54,750 (USD27.08 to USD152.08). Average cost of hospitalization for the 9 patients who had cerebrovascular accidents was NGN116,731.11 ± NGN68,860.97 (USD324.25 ± USD191.28) and expenditures ranged from NGN30,580 to NGN225,530 (USD84.94 to USD626.47). Average cost of hospitalization for the 7 patients who had acute chest syndrome was NGN81,317.14 ± NGN46,899.45 (USD225.88 ± USD130.28) and expenditures ranged from NGN25,530 to NGN132,310 (USD70.92 to USD367.53). Average cost of hospitalization for patients who had priapism was NGN142,462.50 ± NGN99,853.96 (USD396.73 ± USD277.37) and ranged from NGN35,530 to NGN276,530 (USD98.69 to USD768.14).

**Table 4:** pattern of health expenditure of patients

Expenditure type	Range (mean ± SD) (US Dollars)	Total amount (US Dollars)	% of total health expenditure
Investigations	4.7 - 313.9 (27.7 ± 39.3)	5,921	28.5
Cost of drugs	4.2 - 416.7 (25.3 ± 35.0)	6,013	28.9
Hospital utilities	1.5 - 384.9 (28.4 ± 54.1)	8,853	42.6
Grand total		20,787	

**Effects of socio-demographic variables and duration of admission on cost of care:** neither age of child, estimated household income nor social class was associated with cost of care via the one-way ANOVA model (p=0.857, p=0.863 and p=0.397 respectively). Although males incurred higher mean hospital costs than females, this difference was not statistically significant (p=0.374). Patients who spent ≥7 days on admission spent an average total cost of N97,186.26 (USD269.96) more than those who spent <7 days on admission and this difference was significant (p=0.000).

## Discussion

Due to the chronic course of sickle cell disease and the unexpected nature of acute and chronic manifestations, patients and their households are generally exposed to a situation that place huge burdens on their finances which may constitute an impediment to care [2,14,15]. Hence, this population is particularly in great need of universal health coverage through avenues such as prepayment plans offered in health insurance. However, in our study population, only 65 (20.7%) were enrolled in a form of health insurance out of which only 37 (11.8%) used health insurance to make payments at the health facility. Payments were largely out of pocket which have been variously reported to be associated with an increase in the level of disparities that emerge as a consequence of households across various socioeconomic strata struggling to cope with the burden of health expenditures [16-18]. From our study, total expenditures did not depend on social class nor did it depend on household monthly income, households were exposed to similar cost implications irrespective of their socioeconomic status. Some of these households had to depend on contributions from friends and family members as well as borrow in order to shoulder healthcare costs, as was also reported in a previous study [3]. Although there have been efforts by the Nigerian government to ensure wider coverage [6,19], only 22 households (7%) were government beneficiaries with more households enrolled in private insurance. Despite the low level of health insurance enrolment and utilization, majority of the households were aware of health insurance (71.3%) and agreed it was valuable (68.5%). However, fewer households (44.6%) were willing to enrol in a scheme.

Expectedly, awareness significantly decreased among households from lower social classes. Parents in this study showed a high preference for pre-facility treatment (60.8%). This has been reported in earlier studies and has been attributed to an avoidance of being faced with high healthcare costs [20,21]. Home treatment was significantly associated with social class and household income in our study. Majority of the parents in this study highlighted that they knew what medicines to take as part of their reasons for pre-facility treatment. Parents also highlighted issues of distance to the health facility, unfriendly attitudes of professional health care workers, religious/traditional beliefs and use of herbal/alternative medicines such as Agbo as reasons for pre-facility care. Previous studies have highlighted the use of native medicines among respondents even in urban settings [22,23] and children with sickle cell disease are in no way exempted from the adverse consequences of improperly dosed and standardized herbal medications as well as drug-herb interactions that could result from their intake. As was also observed in a previous study [12], average cost of care at our facility was high for a country with a majority of the population earning below one U.S. dollar a day and spending more than 40% of their income to satisfy hunger. Cost of hospitalisation was shown to differ according to clinical presentation, the variety of which can occur in all sickle cell disease patients irrespective of socio-economic background. Our study showed that this cost of hospitalization significantly increased with longer duration of hospitalization usually necessitated by more complicated clinical presentations.

Though actual catastrophic health expenditures (CHE) was not calculated in this study, our findings suggest that when mean of total expenditures at our facility was considered, majority of the households were not earning well enough to be prepared for possible CHE. Hospital utilities constituted a significant part of health expenditures borne by the households. As was highlighted in the study done in Ekiti [7], it is indeed likely that the proportion of households who are at risk of CHEs and who actually incur CHEs are more than we can estimate if the health care costs of the other children in the household and cost of care sought for at other health centres are taken into account. The incidence of CHE reported in Nigeria is generally more than other countries in Africa [7,24,25]. A country´s commitment to healthcare plays a huge role in the reduction of CHEs and the consequent improvement in health outcomes in the population [7]. From the above findings, we propose a more aggressive strategy by the government to health care funding and the achievement of universal health coverage. Particular attention should be paid to populations at risk of fatal adverse health conditions like patients with sickle cell disease which would encourage better access to healthcare facilities and health outcomes. The government, in making efforts in this regard can begin by shouldering the costs of hospital utilities for patients with sickle cell disease as these constituted a significant proportion of health expenditure.

Focused education and awareness of health insurance should be encouraged among parents of children with sickle cell disease to encourage better participation in prepayment plans to forestall development of complications. Also, parents should be educated about the beneficial effects of seeking care at a health facility as opposed to home treatment and other forms of self-medication. More emphasis should be placed on the prevention of comorbidities and complications which would mitigate high health care costs incurred in their management. This study was limited by the fact that it was a hospital-based study and so might not have assessed the burden of care on SCD patients who did not present in hospital settings, indirect costs of care such as time lost from work, costs of transportation e.t.c. were also not taken into account and cost of care sought for outside the hospital setting were not taken into account. We were unable to follow respondents over a one-year period so as to collate more descriptive cost implications of care alongside incidence of all possible comorbidities and complications that a child could experience over a period of time, we only collated incident data.

## Conclusion

Our findings show that health expenditures for the care of children with sickle cell disease was largely out-of-pocket, awareness of health insurance was high however enrolment and utilization was low and there was a high preference for pre-facility treatment among parents. Our findings also show that cost of care for sickle cell disease was high in our facility, varied according to severity of comorbidities and households were exposed to similar cost implications irrespective of socioeconomic status. Duration of hospitalization was significantly associated with higher cost of care. There is therefore need to improve education, awareness and uptake of health insurance for the care of children with sickle cell disease in order to improve health outcomes.

### What is known about this topic

- Nigeria has the highest burden of sickle cell disease globally;
- Payment for healthcare in Nigeria is still largely out of pocket despite WHO recommendations;
- Out of pocket payments create health disparities and poses barriers in the efficient management of patients.

### What this study adds

- Health expenditures for children with sickle cell disease are largely out-of-pocket and despite a high rate of awareness, enrolment and utilization of health insurance is low in our facility;
- There is a high preference for pre-facility treatment in the population;
- The cost of care is high among households of children with sickle cell disease seeking care at the Lagos University Teaching Hospital, varies according to disease severity, significantly increases with increased duration of hospitalization and is so, irrespective of socioeconomic status.
